# Vertically integrated medical education and the readiness for practice of graduates

**DOI:** 10.1186/s12909-015-0514-z

**Published:** 2015-12-21

**Authors:** Marjo Wijnen-Meijer, Olle ten Cate, Marieke van der Schaaf, Chantalle Burgers, Jan Borleffs, Sigrid Harendza

**Affiliations:** 1 Department of Education and Training, Leiden University Medical Center, Leiden, The Netherlands; 2 Center for Research and Development of Education, University Medical Center Utrecht, Utrecht, The Netherlands; 3 Department of Education, Utrecht University, Utrecht, The Netherlands; 4 Center for Innovation and Research in Medical Education, University of Groningen and University Medical Center Groningen, Groningen, The Netherlands; 5 III. Department of Internal Medicine, University Medical Center Hamburg-Eppendorf, Martinistr. 52, 20246 Hamburg, Germany; 6 Department of Medicine, University of California, San Francisco, USA

**Keywords:** Assessment, Entrustable professional activities, Medical education, Vertically integrated curriculum

## Abstract

**Background:**

Medical curricula become more and more vertically integrated (VI) to prepare graduates better for clinical practice. VI curricula show early clinical education, integration of biomedical sciences and focus on increasing clinical responsibility levels for trainees. Results of earlier questionnaire-based studies indicate that the type of the curriculum can affect the perceived preparedness for work as perceived by students or supervisors. The aim of the present study is to determine difference in actual performance of graduates from VI and non-VI curricula.

**Methods:**

We developed and implemented an authentic performance assessment based on different facets of competence for medical near-graduates in the role of beginning residents on a very busy day. Fifty nine candidates participated: 30 VI (Utrecht, The Netherlands) and 29 non-VI (Hamburg, Germany). Two physicians, one nurse and five standardized patients independently assessed each candidate on different facets of competence. Afterwards, the physicians indicated how much supervision they estimated each candidate would require on nine so called “Entrustable Professional Activities (EPAs)” unrelated to the observed scenarios.

**Results:**

Graduates from a VI curriculum received significantly higher scores by the physicians for the facet of competence “active professional development”, with features like ‘reflection’ and ‘asking for feedback’. In addition, VI graduates scored better on the EPA “solving a management problem”, while the non-VI graduates got higher scores for the EPA “breaking bad news”.

**Conclusions:**

This study gives an impression of the actual performance of medical graduates from VI and non-VI curricula. Even though not many differences were found, VI graduates got higher scores for features of professional development, which is important for postgraduate training and continuing education.

## Background

In the past decades, many medical school curricula have been revised so that they represent vertically integrated (VI) programs. A fully vertically integrated undergraduate medical curriculum can be defined by the following four features [1]: provision of early clinical experience; integration of biomedical sciences and clinical cases; progressive increase of clinical responsibility longitudinally and extended clerkships in the final year of medical school [2–5]. One specific aim of vertical integration is to facilitate the transition from medical school to clinical practice and postgraduate training [4]. In earlier studies, we found that, in comparison with those who have followed non-VI programs, graduates of VI curricula appear to make definitive career choices earlier, need less time and fewer applications to obtain residency positions and feel more prepared for work and postgraduate training [6, 7]. Results of other studies indicate that the type of the curriculum can affect the perceived preparedness for work [1, 8–10]. Three of these studies [1, 8, 10] are questionnaire-based analyses of student or faculty perceptions. The fourth study [9] is a focus group study.

To substantiate these impressions there is a need to investigate the actual performance of graduates from VI and non-VI curricula. As known from previous research, two factors contribute to the development of (medical) performance: the amount of knowledge and the amount of experience in practice [11]. Keijers et al. [12] found that students who had followed a VI curriculum had less knowledge of basic medical sciences at graduation in comparison with students from a more conventional, non-VI curriculum. Little is known about the impact of the increased and qualitatively different clinical experience of students in VI curricula. By “hearsay”, the supervisors of postgraduate training programs have much appreciation for the proactive clinical functioning and competences of VI graduates, such as coping with unfamiliar clinical situations. This could be explained by the fact that VI graduates have had more clinical experience during the first years of medical school and have had extended clerkships, giving them the opportunity to see more types of patients. Besides, students get more responsibility during their final clerkships. Numerous studies described the positive influence of these curriculum aspects on learning [13–15].

The purpose of the current study was to determine differences in readiness for clinical practice between graduates from a VI curriculum and those from a non-VI curriculum. More specifically, the research question was: Do graduates from a medical school with a VI curriculum differ from graduates from a medical school with a non-VI curriculum in their competences to cope with unfamiliar clinical situations? This research question was chosen because the clinical context is ever-changing and unpredictable. Coping with real life clinical problems means coping with unfamiliar situations.

The study was carried out among near-graduates from the medical schools in Utrecht and Groningen (The Netherlands) and Hamburg (Germany). The medical schools in Utrecht and Groningen have a vertically integrated curriculum, according to the description above. An important part of the VI curriculum is the degree of responsibility that final year medical students take on. In their role as “semi-physicians” they are expected to bear responsibility for patient care comparable to the responsibility of junior residents [5, 7]. A recent Dutch national review committee judged positive about the increased responsibility in final year clinical clerkships that current medical programs show, based on interviews with physicians involved in residency programs [16]. The reason to compare graduates from these Dutch schools with non-Dutch graduates was that other Dutch schools have developed of are developing VI curricula too. Differences that existed some years ago [7] are gradually disappearing. It was decided to compare with German graduates to maximize chance to find an effect.

The curriculum in Hamburg was not vertically integrated during the execution of this study [17]. In this curriculum there is more emphasis on the acquisition of knowledge, especially in the first two years of the program. In addition, in the medical schools at Utrecht and Groningen, more curriculum time is spent on training communication skills. In both countries, students enter medical school directly after finishing secondary education and in both countries undergraduate medical training lasts six years.

The Educational Commission for Foreign Medical Graduates (ECFMG) of the United States provided us with comparative data of Dutch and German applicants over the period 2002–2011. 1These data showed a pass rate at first attempt for all German applicants for United States Medical Licensing Examination Step 1 Basic Sciences of 76.6 % (*N* = 1861) and 61.1 % for Dutch applicants (*N* = 193). For USMLE Step 2 Clinical Knowledge these figures were exactly similar at 85.5 % (N Germans = 1413; N Dutch = 110); for USMLE Step 2 Clinical Skills they were 84.3 % (N Germans = 1241) and 89.6 % (N Dutch = 86). The numbers are too low to draw firm conclusions, but they align with our prior impression that German curricula focus more on basic science knowledge and Dutch more on clinical skills.

We developed a compentency based assessment procedure, which was validated in a separate study [18], following Kane’s argument-based approach for validation [19, 20]. This assessment procedure was used to test students who followed either a VI or a non-VI curriculum.

## Method

### Procedure

We developed and implemented an authentic performance assessment for medical students near graduation to evaluate their readiness for clinical practice. The key question was whether the graduate can be entrusted with critical clinical activities. These activities were defined as situations that have not necessarily been encountered during clerkships, but require adequate coping by junior residents. The assessment was developed in Utrecht in collaboration with Hamburg. Consequently the assessment was called “Utrecht Hamburg Trainee Responsibility for Unfamiliar Situation Test” (UHTRUST). Because the assessment had taken place in two different countries, all procedures were identically designed in two languages (Dutch and German). During the assessment, candidates were placed in the position of beginning residents on a very busy day: “This is your first day as a resident at a ward which is yet unknown to you. Unfortunately, your supervisor is called away. It is not possible to cancel the patient appointments, so you will be responsible for them, but you can call your supervisor for help whenever you feel the need to.” Detailed information about the assessment procedure and selection of participants and candidates is described in another study, about the validation of this procedure [18].

The assessment consisted of three phases. In the first phase (1 h), the candidates saw five standardized patients at the outpatient department with unusual medical problems. During the second phase (3 h), the candidate had time to gather information on the internet or in pocket books, and to request additional information, e.g. lab results. The candidates had to make a diagnosis for the five patients and to draw up an examination- or treatment plan. During this phase, the candidates also had to face seven realistic distracting tasks, like changes in one of the patient’s condition, questions from nurses or junior students and an urgent organizational problem that had to be solved. The candidates had the opportunity to call their supervisors and halfway there was a meeting planned between candidate and supervisor to discuss the progress. In the third phase (30 min), the candidates reported their differential diagnoses and options for policy or treatment. Table 1 provides a schematic overview of UHTRUST.

**Table 1:** Schematic overview of UHTRUST

		Phase 1		Phase 2		Phase 3	
Activities	Briefing	Short meeting with supervisor	Walk to next location	Collection of diagnostic information about five patients	Walk to next location	Report and discuss examination- and treatment plans	Debriefing
		Consultation of five patients		Seven distracting tasks			
				Halfway meeting with supervisor			
				If needed: calls with supervisor			
				Drawing up management plans			
Duration	30 min	1 h	10 min	3 h	10 min	30 min	30 min
Assessors		Standardized patients		Nurse		Physicians 1 and 2	
				Physicians 1 and 2			

At both locations, pilot assessments were organized a few months before the assessment days. One goal of the pilot was to improve the assessment procedure based on the experiences from the pilot. The other goal was to rehearse the complex organization.

### Assessors

Each candidate was independently assessed on different facets of competence by two physicians, a nurse and five standardized patients (SPs) plus one SP who simulated a relative of one of the patients. One of the physicians also acted as the candidate’s supervisor. The second physician was present all day and listened in to conversations between supervisor and candidate through speaker-amplified cell phones and during the face-to-face meetings. The nurses observed the candidate during the second phase and they deliberately disturbed candidates with distracting tasks. After all observations, physicians were asked to individually indicate how much supervision they estimated this trainee would require on nine so called “Entrustable Professional Activities (EPAs)”, unrelated to the observed scenarios. Prior to the assessment, all assessors received a frame-of-reference training [21]. This training included explanations about the use of scoring forms and the impact of scoring biases.

### Comparison the groups of assessors

Because the Dutch candidates were assessed by Dutch assessors and the German candidates by German assessors, we wanted to know whether there were systematic differences in scoring between the groups of assessors [22]. Prior to the actual assessment, both groups of physicians and SPs watched the same video recording of one Dutch and one German candidate who had participated in the pilot of the UHTRUST assessment. Both candidates had been selected at random. We asked the assessors to judge both candidates on the FOC and EPA scoring forms to be used during the assessment days. No statistically significant differences between their mean scores (Mann-Whitney U) was found. The consistency among the physicians’ scores was checked by calculating Jury alpha, for both the total group of physicians and the Dutch and German group separately. In all cases, the internal consistency was very high: Jury alpha varied from .96 to .98. For the nurses, it was not possible to compare groups, because of the small population. For five of seven aspects of the CARE questionnaire, the German SPs gave significantly higher scores in comparison with the Dutch SPs (*p* < .05). This was the case for both candidates. The consistency between the scores of the SPs at either site was very high (Jury alpha is .98).

### Participants

During the assessment days in July and August of 2011, 59 candidates participated: 23 from Utrecht, 7 from Groningen (The Netherlands) and 29 from Hamburg (Germany). The candidates from Utrecht and Groningen participated in the assessment on the same day, which took place in Utrecht. All candidates had nearly graduated from medical school at the time of the assessments. The Dutch students were two weeks before graduation at the moment of the assessment, and the German students three months. This difference is based on the fact that we did not want to assessment day scheduled close to the final knowledge exam in Germany, to prevent possible bias. They participated voluntarily and they presented themselves in reaction to announcements. At both locations, ten physicians and four nurses were involved. Furthermore, in Utrecht six standardized patients (SPs) and in Hamburg 18 SPs participated. The difference between the numbers of SPs is caused by the fact that in Utrecht all 30 times the six roles were played by the same SPs, while in Hamburg for practical reasons every role rotated between three SPs. The physicians and nurses were invited to participate, based on their clinical experience and experience with supervising trainees.

### Instruments

The physicians completed three kinds of scoring forms for each candidate. One scoring form included seven so called facets of competence (FOCs) that are a key in making entrustment decisions by supervisors about residents (see Table 2). These facets had been developed during a Delphi study among physician supervisors [23]. For each FOC, the candidates were scored on a 3-point Likert scale of 1 (weak) to 3 (good) for each of five different patient cases. Next to this, the assessors gave an overall score for each FOC on a 5-point Likert scale, from 1 (very weak) to 5 (very good). The second questionnaire consisted of nine so called “Entrustable Professional Activities” (EPAs), tasks that are suitable to entrust to a trainee once sufficient capability is attained for unsupervised practice [24] (see Table 4). The physicians were asked to indicate on a 5-point scale how much supervision they think that the candidate would need for these EPAs, which were different from the actually observed activities (1 = he/she is not able to do this; 2 = he/she is able to do this under direct supervision; 3 = he/she is able to do this if supervision is available; 4 = he/she is able to do this independently; 5 = he/she is able to supervise others in performing this activity) [25].

**Table 2:** Scoring “facets of competence” by physicians (5-point scale; mean score over two assessors)

Facet of competence	Dutch (VI curriculum); *N* = 30		German (non-VI curriculum); *N* = 29	
	M	SD	M	SD
1. Scientific and empirical grounded method of working	3.23	1.01	3.22	0.94
2. Knowing and maintaining own personal bounds and possibilities	3.32	0.75	3.14	1.01
3. Teamwork and collegiality	3.34	0.61	3.50	0.93
4. Verbal communication with colleagues and supervisors	3.50	0.94	3.50	0.93
5. Responsibility	3.38	0.75	3.28	1.12
6. Safety and risk management	3.02	0.83	3.24	0.96
7. Active professional development^a^	3.55	0.77	2.81	1.14

^a^Mann-Whitney test: *U* = 278, *z* = -2.41, *p* = .02, *r* = .31

The third form was a so called “Post-Patient Encounter Form” (PPEF), based on Durning’s Post-Encounter Form [26]. The candidates summarised on this form for each patient case the most important problems, differential diagnoses and a proposal for treatment. The assessing physicians scored these aspects on a 5-point Likert scale from 1 (below expectations) to 5 (above expectations).

The fourth scoring form was completed by the nurses and contained six FOCs, similar to six of the seven FOCs scored by the physicians (see Table 3). For each FOC, the candidates were scored by the nurse on a 3-point Likert scale of 1 (weak) to 3 (good) for their performance regarding five different disturbances. Additionally, the nurse gave an overall score for each FOC on a 5-point Likert scale, from 1 (very weak) to 5 (very good).

**Table 3:** Scoring “facets of competence” by nurses (5-point scale)

Facet of competence	Dutch (VI curriculum); *N* = 30		German (non-VI curriculum); *N* = 29	
	M	SD	M	SD
1. Scientific and empirical grounded method of working	3.10	0.85	3.08	0.63
2. Knowing and maintaining own personal bounds and possibilities^a^	3.13	0.63	3.46	0.71
3. Teamwork and collegiality^b^	3.17	0.65	3.58	0.64
4. Verbal communication with colleagues and supervisors	3.10	0.85	3.17	0.76
5. Responsibility	3.27	0.69	3.25	0.85
6. Safety and risk management	2.93	0.98	3.20	0.91

^a^Mann-Whitney test: *U* = 487, *z* = 2.08, *p* = .04, *r* = .28

^b^Mann-Whitney test: *U* = 514, *z* = 2.25, *p* = .02, *r* = .30

The SPs completed the so called CARE-questionnaire, a validated instrument consisting of 10 questions to measure consultation skills and empathy [27]. The SPs scored the candidates on a 5-point scale (1 = poor, 2 = fair, 3 = good, 4 = very good, 5 = excellent). The questionnaire included three questions about making action plans together with the patient. Because the candidates in this assessment did not make any action plans, only seven questions of the questionnaire were applicable in this study (see Table 5). At the German site, a different 5-point scale was used to score the CARE-questionnaire (1 = not at all applicable, 2 = hardly applicable, 3 = partially applicable, 4 = largely applicable, 5 = fully applicable). To correct the differences between the two scales, the scores were transformed into a 3-point scale (1 = 1 of the Dutch and 1/2 of the German questionnaire; 2 = 2 Dutch and 3 German; 3 = 3/4/5 Dutch and 4/5 German).

Finally, the candidates completed the NEO-FFI personality test [28]. This is a validated test containing 60 items on a point 5-point Likert scale (from 1 = totally disagree to 5 = totally agree), measuring five personally dimensions: extraversion, agreeableness, conscientiousness, neuroticism and openness to experience.

### Analyses of the data

Mann-Whitney tests were computed to compare the scores of the candidates from the VI and non-VI curriculum. Chi-square were conducted tests to compare the results of the personality tests of the two groups of candidates. To compare the scores given by Dutch and German assessors, Mann-Whitney tests were used and the degree of consistency among the assessors was calculated with Jury alpha. Not all candidates had completed all PPEF forms. Pearson correlation coefficients were calculated between the number of completed PPEFs and the scores on FOCs and EPAs, as we suspected that the number of completed PPEFs could be indicative of the candidate’s proficiency.

### Ethics

Ethical approval for the Dutch part of the study was obtained from the NVMO Ethical Review Board. For the German part, ethical approval was obtained from the State of Hamburg Physicians’ Ethics Board. Written informed consent for participation was obtained from all participants.

## Results

### Background information regarding the candidates

In the Dutch group 20 (67 %) candidates and in the German group 22 (76 %) candidates were female. These percentages approximately reflect the gender distribution of the total groups of medical graduates. Dutch candidates (M = 24.4 year) were younger than the German candidates (M = 26.0 year) based on independent samples 2-tailed *T*-test (T(57) = 4.65, *p* = .00, *r* = .47). No significant correlations between age and scores were found. Hence, it was assumed that this difference in age did not affect the results. Based on the NEO-FFI personality test, there were no differences between the two groups regarding the dimensions neuroticism, extraversion and conscientiousness. The Dutch candidates scored higher on the dimension “openness to experience” (*χ*^2^ (8, 59) = 17.74, *p* = .02). The German candidates had a significantly higher score on the dimension agreeableness (*χ*^2^ (7, 59) = 15.02, *p* = .04).

### Facets of competence

Table 2 shows the mean scores of the physicians on the seven FOCs for the two groups of candidates. Regarding the FOC “active professional development” the Dutch candidates (M = 3.55; SD = .77) received higher scores than the German candidates (M = 2.81; SD = 1.14; *U* = 278; *z* = -2.41; *p* = .02, *r* = .31).

Table 3 compares the mean scores for the two groups of candidates given by the nurses. There is a significant difference between the groups regarding the FOC for “knowing and maintaining own personal bounds and possibilities” (Mann-Whitney *U* = 487; *z* = 2.08; *p* = .04, *r* = .28). German candidates (M = 3.46, SD = .71) had higher scores on this FOC than the Dutch candidates (M =3.13; SD = .63). This is also the case for “teamwork and collegiality” (Dutch candidates: M =3.17; SD = .65; German candidates: M =3.58; SD = .64; *U* = 514; *z* = 2.25; *p* = .02, *r* = .30).

### Entrustable professional activities

Table 4 shows the mean scores for the estimation of required supervision about the various EPAs for the two groups. As can be seen, statistically significant differences for two EPAs were identified. For “breaking bad news” the German group (M = 3.10; SD = .54) scored higher than the Dutch group (M = 2.62; SD = .70; Mann-Whitney *U* = 617; *z* = 2.85; *p* = .00, *r* = .37). In contrast, the Dutch candidates (M = 3.70; SD = .53) received higher scores for “solving a management problem” than the German candidates (M = 3.19; SD = .75; Mann-Whitney *U* = 265; *z* = 2.67; *p* = .01, *r* = .35).

**Table 4:** Scoring “Entrustable professional activities” (EPAs) by physicians (5-point scale; mean score over two assessors)

EPA	Dutch (VI curriculum); *N* = 30		German (non-VI curriculum); *N* = 29	
	M	SD	M	SD
1. Emergency assistance with acute cardiac failure	2.33	0.53	2.36	0.57
2. Handling a patient complaint	2.70	0.92	2.86	0.63
3. Pre-operative information and consent	3.42	0.54	3.43	0.59
4. Breaking bad news^a^	2.62	0.70	3.10	0.54
5. Clinical reasoning under time pressure	2.38	0.57	2.38	0.56
6. Solving a management problem^b^	3.70	0.53	3.19	0.75
7. Suspicion of self-induced disease	2.93	0.57	2.91	0.44
8. Handling of a seriously ill patient	2.40	0.58	2.21	0.68
9. Interaction with a consultant	3.23	0.60	3.28	0.64

^a^Mann-Whitney test: *U* = 617, *z* = 2.85, *p* = .00, *r* = .37

^b^Mann-Whitney test: *U* = 265, *z* = 2.67, *p* = .01, *r* = .35

### Post-patient encounter forms

The candidates were instructed to complete a Post-Patient Encounter Form (PPEF) for each of the five patient cases prior to the reporting phase, with a summary of the most important problems, differential diagnoses and a proposal for treatment. Within the Dutch group 95 % of the PPEFs were completed, the German group only completed 67 %. Pearson correlation coefficients were calculated between the number of completed PPEFs and the scores on FOCs and EPAs. Significant correlations for five FOCs and two EPAs were identified, which led us to believe that the number of completed PPEFs is an indicator of proficiency. This would mean that within the German group the PPEF responders make a positively biased subgroup. Because of these findings, it was decided not to compare scores on the PPEFs between the two groups.

### Standardized patients: CARE-questionnaire

The mean scores on the CARE-questionnaire are presented in Table 5. The German candidates received significantly higher scores for “making you feel at ease” (M = 2.86; SD = .18; *U* = 639.50; *z* = 3.22; *p* = .00, *r* = .42) and “really listening” (M = 2.88; SD = .12; *U* = 573.50; *z* = 2.21; *p* = .03, *r* = .29), in comparison with the Dutch candidates (M = 2.69; SD = .21 and M = 2.79; SD = .17 respectively). For the aspect “being positive” the Dutch candidates had significantly higher scores (M = 2.80; SD = .19) than the German candidates (M = 2.58; SD = .36; *U* = 279; *z* = -2.43; *p* = .02. *r* = .32).

**Table 5:** Scoring statement CARE-questionnaire by standardized patients (3-point scale; mean score across five SPs and one SP relative)

How was the doctor at…	Dutch (VI curriculum); *N* = 30		German (non-VI curriculum); *N* = 29	
	M	SD	M	SD
1. Making you feel at ease^a^	2.69	0.21	2.86	0.18
2. Letting you tell your story	2.83	0.17	2.89	0.14
3. Really listening^b^	2.79	0.17	2.88	0.12
4. Being interested in you as a whole person	2.68	0.20	2.55	0.28
5. Fully understanding your concerns	2.72	0.26	2.74	0.23
6. Showing care and compassion	2.73	0.25	2.76	0.26
7. Being positive^c^	2.80	0.19	2.58	0.36

^a^Mann-Whitney test: *U* = 639.50, *z* = 3.22, *p* = .00, *r* = .42

^b^Mann-Whitney test: *U* = 573.50, *z* = 2.21, *p* = .03, *r* = .29

^c^Mann-Whitney test: *U* = 279, *z* = -2.43, *p* = .02, *r* = .32

## Discussion

Our study was designed to test the differences in readiness for clinical practice between graduates from VI and non-VI curricula. Results of earlier studies indicate that the type of curriculum can affect the preparedness for practice [1, 6–10, 29, 30]. All of these studies are analyses based on questionnaires or focus groups of student [6–10, 29, 30] or supervisor perceptions [1]. In one study, also the differences in knowledge acquisition and clerkships grades were described [30]. To substantiate these findings, the actual performance of graduates from VI and non-VI curricula was investigated by means of an authentic assessment. In a separate study, a validity argument for the UHTRUST assessment procedure was provided. The results of this parallel study indicate that most validity assumptions were defendable with accurate and often parallel lines of backing and that UHTRUST can be used to assess the readiness for clinical practice of medical graduates [18].

EPAs were used to create sense of trustworthiness of the candidates. Entrustment decisions are based on a variety of observations that enable inferences about required level of supervision for future executions of these activities. This study did just that. In educational programs, the inferences will usually be based on multiple observations of the execution of an EPA, but the inference is not conceptually different. It is the estimation that the risks by permitting to execute the EPA unsupervised in future cases is manageable [31, 32].

Contrary to our expectations, only few differences between the two groups of candidates existed despite large differences in the curriculum they had followed. One finding is that, based on the judgments of the physicians, the candidates from a VI curriculum scored better on the facet of competence “active professional development”. The complete description of this FOC is: “The physician aims for quality and professional development by means of a critical attitude towards himself and his environment, study, self-assessment, reflection, asking for feedback and setting and achieving learning goals. S/he reacts to criticism constructively and is aware of his/her own responsibility regarding his/her own abilities”. This FOC is relevant for the continuing education of physicians, which requires that medical graduates are capable of setting their own learning goals, receiving feedback and reflection [33].

Next to this, the VI candidates received higher scores for the EPA “solving a management problem” and for “being positive” at the CARE-questionnaire for standardized patients. On the other hand, the non-VI candidates were judged better on the FOCs “teamwork and collegiality” and “knowing and maintaining own personal bounds and possibilities” by the nurses and on the EPA “breaking bad news” by the physicians. They also had higher scores for the aspects “making you feel at ease” and “really listening” in the CARE-questionnaire. Because of the amount of tests, the discovered differences may be due to chance. Reaffirmation of the results in future studies is therefore desirable.

Based on the NEO-FFI personality test, the Dutch and German candidates differed on the dimensions “openness to experience” and “agreeableness”. Based on a meta-analysis of comparison studies, Barrick et al. [34] concluded that only the dimensions of neuroticism and conscientiousness can be used to predict job performance. Cave et al. [10] found that junior doctors with high scores on the dimensions extraversion and conscientiousness feel better prepared for clinical practice, in contrast to junior doctors with high scores on neuroticism. Based on the results of these studies it can be assumed that the differences in personality found between the two groups of candidates did not affect the results of our study.

This study has a number of limitations. One limitation is that the two groups of candidates are relatively small and from different countries with, possibly cultural, differences. Another limitation related to this, is the fact that the candidates were assessed by physicians, nurses and standardized patients from their own country. There was no possibility to employ neutral assessors for all candidates. To reduce this disadvantage, we compared scoring standards of both groups of assessors, by letting them score a Dutch and a German candidate who had been video recorded during a pilot assessment. No differences between assessors from different countries were found. Unfortunately, standards among nurses could not be compared because of the low number of raters. German SPs gave higher scores than Dutch SPs on the videotaped examples, which makes it difficult to interpret the results of these parts of the study. Finally, we did not have full control over the populations because the candidates signed up for participation voluntarily, which may have affected the results.

Several authors have argued that it is difficult to investigate the impact of different curricula on the readiness for practice of medical graduates by means of experimental research [35–38]. One argument for this statement is that the many components of a curriculum form a complex entity, which makes it difficult to determine the influence of the specific features, which differ from each other. Additionally, there are many differences in background and characteristics among the students of the same medical school. Nevertheless, we expected to find differences between the performances of the VI and non-VI graduates, because the two curricula differ in many respects from each other. Especially the differences in amount of clinical experience and the responsibility for patient care of final year medical students are believed to have a big impact on the development to junior doctors [11, 13, 15]. In addition, ECFMG figures over the period 2002–2011 give some information to expect a difference, if carefully evaluated.

Despite the limitations, the current study contributes to the existing literature. We spent maximum effort to construct a valid assessment procedure [18] that should allow detecting differences, if they exist. This study has been unable to demonstrate that vertically integrated curricula prepare their graduates better for clinical practice. But there is no reason to jump to the conclusion that the trend towards vertical integration is not the right direction. First, because numerous educational theories about expertise development suggest that a positive effect of vertical integration is to be expected [11, 14, 39–41]. Second, because other studies detected evidence for these theories [1, 6–10].

There are several possible explanations for the fact that only few differences between graduates from VI and non-VI curricula were found. One possibility is that the populations are unequal and not random. In addition, it is possible that despite the preparatory training, assessors were focused on different aspects of the performance because of their personal experiences and background [42, 43]. To reduce these differences, the frame-of-reference training [21] possibly needs improvement or extension. The differences between the groups of assessors are conceivably strengthened because they are from different countries with different cultures at the clinical workplace.

## Conclusions

Our newly developed assessment for medical students at the time of their graduation gives a first impression of their actual competency-based performance. Authentic assessments like UHTRUST, based on realistic situations can provide students with relevant feedback. Even though we did not find many differences, VI graduates got higher scores for features of professional development, which is important for postgraduate training and continuing education. Similar studies with this new competency assessment are needed within one country, before the difference in performance of VI and non-VI graduates is more clearly understood.

## Footnotes

1 We thank Dr John R Boulet, Chair Internal Research and Data Review Committee Educational Commission for Foreign Medical Graduates for his help in providing these data
